# The completeness of adverse drug reaction reports in South Africa: An analysis in VigiBase^®^

**DOI:** 10.4102/phcfm.v15i1.3659

**Published:** 2023-01-18

**Authors:** Mafora F. Matlala, Martha S. Lubbe, Hanlie Steyn

**Affiliations:** 1 Medicine Usage in South Africa, Faculty of Health Sciences, North-West University, Potchefstroom, South Africa; 2 Pharmacovigilance Unit, South African Health Products Regulatory Authority, Pretoria, South Africa

**Keywords:** pharmacovigilance, adverse drug reactions, reports, completeness, South Africa, individual case safety report

## Abstract

**Background:**

Spontaneous reporting is regarded as a cornerstone of pharmacovigilance (PV) but presents many limitations, including varying quality and completeness of information, which is essential for causality assessment.

**Aim:**

This study aimed to evaluate the completeness of adverse drug reaction (ADR) reports in South Africa based on the vigiGrade completeness score.

**Setting:**

The South African Health Products Regulatory Authority (SAHPRA).

**Methods:**

A cross-sectional, descriptive study of all reports received by SAHPRA and submitted to VigiBase^®^ in 2017 was conducted. A report with a vigiGrade score > 0.8 is considered well-documented.

**Results:**

The mean completeness score for the 8438 reports received was 0.456 (s.d. = 0.221). Only 11.3% of reports had a completeness score > 0.8. The completeness of reports submitted by consumers professionals did not significantly differ from reports by physicians, pharmacists or other healthcare professionals (*d* ≤ 0.2). Reports of reactions that resulted in death (*M* = 0.572, s.e. = 0.007), disability (*M* = 0.491, s.e. 0.033) or were life threatening (*M* = 0.474, s.e. = 0.013) had a medium to large practically significant effect (0.5 ≥ *d* ≤ 0.8) on the completeness score compared with reports of congenital anomaly (*M* = 0.348, s.e. = 0.089).

**Conclusion:**

The completeness of reports submitted by consumers is comparable to those submitted by healthcare professionals. The completeness of reports was low and multiple measures to improve reporting are recommended.

**Contribution:**

This study describes the completeness of ADR reports in South Africa and the results can be used to improve training.

## Introduction

Pharmacovigilance (PV) is defined as ‘the science and activities relating to the detection, assessment, understanding and prevention of adverse drug reactions (ADRs) or other drug-related problems’.^1^ Once approved for marketing, drugs are continuously monitored for ADRs to ensure a favourable risk-benefit ratio. Post-marketing safety monitoring mainly occurs via spontaneous reporting of ADRs by healthcare professionals (HCPs) or consumers to either the marketing authorisation holder (MAH) or the local regulatory authority.^2^ According to Moore et al., spontaneous ADR reports were the primary source of scientific information used by the Food & Drug Administration (FDA) to make regulatory decisions during 2009 and formed the basis of 57% new regulatory actions and 76% new boxed warnings.^3^ Further analysis by Lester et al. indicated that spontaneous reports were the typical evidence source contributing to label changes by the FDA in 2010.^4^ The three most prevalent evidence sources that contributed to a label change were spontaneous reports (52%), clinical trials (16%) and pharmacokinetic studies (11%).^4^

Even though spontaneous reporting is a cornerstone of PV, with many significant strengths, including early detection of safety alerts, it also has many limitations. These limitations include low reporting rates, varying quality, completeness and accuracy of the provided information.^5^ The incompleteness of spontaneous reports poses a great concern because of causality assessment, ‘the process during which the level of probability between the ADR and the role of the suspect drug as the causative agent is determined’, depends on this parameter.^6^ The quality of the information provided in case reports influences the strength of the causal relationship between the suspect drug and the reaction.^7^

In 1965, the English epidemiologist Sir Austin Bradford Hill (1897–1991) established a group of minimal criteria necessary to provide adequate evidence of a causal relationship between incidence and a consequence. The Bradford Hill criteria, also known as Hill’s criteria for causation, assess causality from multiple information sources using the following parameters: ‘strength of association, temporality, consistency, theoretical plausibility, coherence, specificity in the causes, dose–response relationship, experimental evidence and analogy’.^8^

Bergvall et al. have further indicated that the important information required for causality assessment leading to signal detection includes time-to-onset of the ADR, patient age and sex, outcome and indication for treatment.^9^

Durrieu et al. revealed that only 12.7% of reports submitted by general practitioners were classified as well-documented and concluded that it is essential to promote quality data to optimise signal detection.^10^ Sánchez-Sánchez et al. evaluated the completeness of suspected ADR reports submitted to the Mexican National Pharmacovigilance Centre in 2008 and indicated that most reports contained incomplete information. About 40% of the reports contained the date of suspected ADR; however, treatment start dates were unknown, making it impossible to determine the time-to-onset of the reaction.^11^

The Uppsala Monitoring Centre (UMC), the World Health Organization (WHO) collaborating centre for international drug monitoring, is responsible for signal detection and dissemination of drug safety issues. Therefore, the UMC holds and maintains the VigiBase^®^ (Uppsala, Monitoring Centre, Uppsalla, Sweden), the WHO global database of individual case safety reports (ICSRs).^12,13^

Following the low levels of completeness of ICSRs submitted by regulatory authorities to VigiBase^®^, a tool used to measure the level of completeness of ICSRs in VigiBase^®^, known as vigiGrade, was developed by the UMC.^9,14^ The Uppsala Monitoring Centre automatically calculates the vigiGrade completeness score for each report committed to VigiBase^®^ and reports with a completeness score > 0.8 are considered well-documented.^9^ National pharmacovigilance centres can view the completeness score per report through VigiLyze^®^ (Uppsala Monitoring Centre, Uppsala, Sweden), the search and analysis software that enables exploration of VigiBase^®^. Bergvall et al. have reported an average completeness score of 0.45 for all reports in VigiBase^®^, while only 13% of the reports were considered well-documented.^9^ Measuring and communicating the completeness to PV centres is the first step to help improve the quality of reports.^9^ Having been a member of the WHO Programme for International Drug Monitoring (WHO-PIDM) since 1992,^15^ the South African Health Products Regulatory Authority (SAHPRA) submits ADR reports to VigiBase^®^. No study has been conducted to evaluate the completeness of South African ADR reports submitted to VigiBase^®^. During 2015, SAHPRA acquired the VigiFlow^®^ system (Uppsala Monitoring Centre, Uppsala, Sweden), which supports the domestic collection and processing of ICSRs, before being committed to the VigiBase^®^. It was used as a pilot project during the year 2016. The year 2017 was the 1st year in which all ADR reports received by the SAHPRA were entered into the system. This study was undertaken between 2018 and 2021 and during study protocol development in 2018 and data analysis in 2019 only the 2017 data were available for analysis.

Adverse drug reactions can be reported to SAHPRA on the ADRs & Quality Problem Reporting Form (yellow form), Council for International Organizations of Medical Sciences (CIOMS) form, the Adverse Events Following Immunisation (AEFI) form, and other forms used in South African public health programmes. Reports can also be submitted via the Med Safety App, e-reporting portal on the SAHPRA website, email or telephonically via a hotline. This study aims to evaluate the completeness of reports submitted to SAHPRA and committed to VigiBase^®^ for the year 2017 based on the vigiGrade completeness score.

## Research methods and design

### Study design

A cross-sectional, descriptive study of all ICSRs received by the SAHPRA and submitted to VigiBase^®^ during 2017 was conducted.

### Setting

The study was conducted in the Pharmacovigilance Unit of the SAHPRA.

### Study population

Adverse drug reaction reports were captured into the VigiFlow^®^ system, which supports the domestic collection and processing of ICSRs, before being committed to the VigiBase^®^. For MAHs, reporting of serious ADRs is mandatory, and these reports should be sent to SAHPRA within 15 calendar days of receipt.^16^ Reports were received from different reporter categories as per their qualifications, namely physicians, pharmacists, consumers or non-HCPs, lawyers and other HCPs (e.g. nurses). VigiLyze^®^ exports do not indicate the sender of the report (i.e. MAHs, clinical trials, HCPs or non-HCPs) or the report reporting method (e.g. Med Safety App, e-reporting or paper form).

### Data collection

The ICSRs were extracted from VigiBase^®^ on 11 June 2019 with the search and analysis software known as VigiLyze^®^. VigiLyze^®^ exports contain information about each case report as well as complete information on medications and the ADR. Demographic variables of the patients included age and sex. Age groups: neonates (0–27 days), infants (28 days – 23 months), children (2–11 years), adolescents (12–18 years), adults (19–64 years) and elderly (> 65 years). The country of origin for all reports analysed was South Africa.

The seriousness of the reactions was classified as resulting in death, life-threatening, disabling, hospitalisation or prolongation of hospitalisation, congenital anomaly and other medically important event. The outcome of the reaction was categorised as died, recovered, not recovered, recovering and unknown.

The suspect and concomitant medicines were classified according to the Anatomical Therapeutic Chemical (ATC) classification system,^17^ in which the ‘active substances are divided into different groups according to the organ or system on which they act and their therapeutic, pharmacological and chemical properties’. Drugs are classified in groups at five different levels, and in this study, medicines were evaluated according to level 1 within 14 main ATC groups. Adverse drug reactions were classified according to the Medical Dictionary for Regulatory Activities (MedDRA^®^) preferred term and System Organ Class.^18^

The start date of the drug and the onset date of the reaction were used to calculate the time-to-onset of the reaction. The completeness score for each report is calculated by UMC and can be viewed in VigiLyze^®^. The vigiGrade completeness score is restricted to important information for causality assessment and is expected to be present on most reports.^9^ The vigiGrade completeness score evaluates the dimensions of the report rather than the specific elements included in it. Three levels of importance are identified, namely:

[*E*]ssential (information without which reliable causality assessment is impossible), important (information without which reliable causality assessment is very difficult), and supportive (information that is valuable but without which reliable causality assessment can still be performed).^9^

If a dimension is not reported, the completeness score suffers a penalty. Imprecise information is penalised less compared with the total omission of information. The more important the information is in the clinical assessment of a drug reaction relation, the higher the penalty factor.^9^ The vigiGrade completeness score (*C*) starts at 1 for reports containing all important variables for causality assessment. The vigiGrade penalties are defined as indicated in Table 1.^9^

**TABLE 1:** Overview of the dimensions and associated penalties accounted for in the vigiGrade completeness score.

Dimension	Considerations	Penalty %
Time-to-onset	Imprecise information penalised if there is ambiguity as to whether the drug preceded the adverse event, by 30% if the uncertainty exceeds one month, 10%	50
Indication of use	The penalty imposed if the information is missing or cannot be mapped to standard terminologies such as ICD or MedDRA^®^	30
ADR outcome	None	30
Sex	‘Unknown’ treated as missing	30
Age	Age ‘unknown’ treated as missing 10% penalty imposed if the only age group is specified	30
Dose	None	10
Reporter country	Supportive in causality assessment because medical practice and adverse reaction reporting vary between countries	10
Primary reporter	Supportive in causality assessment because the interpretation of reported information may differ depending on the reporter’s qualification ‘Unknown’ penalised as missing information, but ‘other’ not penalised	10
Report type	None	10
Comments	Uninformative text snippets excluded	10

*Source*: Bergvall T, Norén GN, Lindquist M. vigiGrade: A tool to identify well-documented individual case reports and highlight systematic data quality issues. Drug Saf. 2014;37(1):65–77. https://doi.org/10.1007/s40264-013-0131-x

ADR, adverse drug reaction; ICD, MedDRA^®^, Medical Dictionary for Regulatory Activities.

vigiGrade classifies a report with a final score of *C* ≥ 0.8 as well-documented.^9^ This limit allows for lack of information on two supportive variables, but with a penalty of 50% on the essential variables and 30% on important variables.^9^ The vigiGrade completeness score (*C*) in categories < 0.5, 0.5 ≥ 0.8 and > 0.8 has been reported.

### Data analysis

The Medical Dictionary for Regulatory Activities (MedDRA^®^ version 22.0) de-duplicated data set was exported to Microsoft Excel and was analysed using the Statistical Package for Social Sciences (SPSS^®^) version 25.0.^19^ Data were first analysed using descriptive statistics, where frequencies and percentages were calculated for all variables. Measures of central tendency for continuous variables were displayed as means and corresponding standard deviation or error of the mean. The one-way analysis of variance (ANOVA) was used to compare the continuously distributed mean completeness scores between different categories of each variable analysed. All statistical tests were conducted at the 5% significance level, and Cohen’s *d* was calculated as a measure of effect size. Cohen’s *d* guideline: small effect ≤ 0.2; medium effect > 0.2 and < 0.5; large effect ≥ 0.5 and < 0.8; practical significant ≥ 0.8.

### Ethical approval

This study was approved by the North-West University Health Research Ethics Committee (No. NWU-00012-19-S1) and goodwill permission was obtained from the South African Health Products Regulatory Authority (SAHPRA). This study was performed in line with the principles of the Declaration of Helsinki.

## Results

South African Health Products Regulatory Authority received 8438 ICSRs that were committed to VigiBase^®^ from 01 January 2017 to 01 December 2017. These reports contained 29 826 drug reaction pairs, of which 20 438 were for suspected medicines and 9388 for concomitant medicines. The mean vigiGrade completeness score for the 8438 reports was 0.456 (s.d. = 0.221). As a result of the multiaxiality of MedDRA^®^ (The International Council for Harmonisation of Technical Requirements for Pharmaceuticals for human use [ICH], Herndon, Virgina, United States of America), reports can contain more than one suspect or concomitant medicine or more than one reaction.

### Incomplete data fields

Table 2 displays the frequencies and percentages of incomplete data fields. Variables that are valuable for causality assessment and penalised if absent for vigiGrade completeness score calculation are indicated.

**TABLE 2:** Incomplete data fields.

Reports (*N* = 8438)			Drug-reaction pairs (*N* = 29 826)			Suspected drug-reaction pairs (*N* = 20 438)		
Data field	Missing		Data field	Missing		Data field	Missing	
	*n*	%		*n*	%		*n*	%
Seriousness	3724	44.13	Dose	15 011	50.30	Indication	3278	16.00
Outcome	923	10.94	-	-	-	Action taken with the drug	3075	15.00
Reporter qualification	230	2.73	-	-	-	Start date of the drug	10 754	52.60
Sex	421	4.99	-	-	-	Onset date of the reaction	1062	5.20
Age group	2097	24.85	-	-	-	Time-to-onset	10 795	52.82

Time-to-onset of the ADR, which is a crucial factor needed for causality assessment, could not be calculated for 52.82% (*n* = 10795) of suspected drug-reaction pairs. The vigiGrade completeness score applies a 50% penalty if time-to-onset is missing in the report. The ADR outcome was missing on 10.94% (*n* = 923) of the reports and suffered a 30% vigiGrade penalty. Where the event outcome was reported, it was reported as unknown on 63.32% (*n* = 5343) of the reports. The indication for using the suspected drugs was missing on 16% (*n* = 3278) of reports, resulting in a 30% vigiGrade penalty. Among the 8348 reports analysed, the age and sex were incomplete in 2097 (24.8%) and 421 (4.9%) reports, respectively, and suffered a 30% vigiGrade penalty. The drug dose was incomplete for 50.30% (*n* = 15 011) of the drug-reaction pairs and suffered a 10% penalty. Concerning ‘action taken with the drug’, 15% (*n* = 3075) of reports did not have this variable completed.

### Reporter categories

Table 3 indicates the number of reports and the mean completeness scores for the reports submitted by each reporter category, according to their qualifications. Reporter qualification was not indicated in 230 (2.73%) reports received.

**TABLE 3:** Completeness scores for different reporter qualifications.

Reporter qualification	Number of ADR reports		*M*	s.e.	95% CI	Number of ADR reports per completeness score category							
						< 0.5		0.5 ≥ 0.8		> 0.8		Total	
	*n*	%				*n*	%	*n*	%	*n*	%	*n*	%
Consumer or non-HCPs	2412	28.58	0.436	0.004	0.427 – 0.444	1634	67.7	511	21.2	267	11.1	2412	100
Lawyer	1	0.01	0.284	0.219	−0.145 – 0.713	1	100	0	0	0	0	1	100
Other HCPs	2175	25.78	0.498	0.005	0.489 – 0.507	1165	53.6	697	32.0	313	14.4	2175	100
Pharmacist	365	4.33	0.480	0.011	0.458 – 0.503	197	54.0	113	31.0	55	15.1	365	100
Physician	3255	38.58	0.450	0.004	0.442 – 0.457	1985	61.0	959	29.5	311	9.6	3255	100
All reports	8438†	100	0.456	-	-	5178	61.4	2307	27.3	953	11.3	8438†	100

ADR, adverse drug reaction; s.e., standard error of the mean; CI, confidence interval; M, mean; HCP, healthcare professionals.

† , Reporter qualification was not reported on 230 (2.73%) of all reports.

Reports were submitted by physicians (38.58%; *n* = 3 255), consumers or non-HCPs (28.58%; *n* = 2412), other HCPs (25.78%; *n* = 2175), pharmacists (4.33%; *n* = 365) and one lawyer (0.01%; *n* = 1). The mean completeness scores for the different reporter categories varied between *M* = 0.284, s.e. = 0.219 for the lawyer and *M* = 0.498; s.e. = 0.005 for other HCPs. The one-way ANOVA test showed a statistically significant difference in the completeness scores between reporter categories, *F* (5, 8432) = 38.738, *p* ≤ 0.001. The completeness score of reports submitted by pharmacists and other HCPs showed practically significant differences compared with the lawyer (*d* ≥ 0.8). Reports that did not indicate the reporter qualification suffered a 10% vigiGrade penalty and had a medium to large practically significant effect (0.5 ≥ *d* ≤ 0.8). The completeness of reports submitted by consumers or non-HCPs did not significantly differ compared with reports submitted by physicians, pharmacists or other HCPs (*d* ≤ 0.2). Only 953 (11.3%) of the reports received had a completeness score *C* > 0.8 and can be classified as well-documented and 5178 (61.4%) of the reports received a completeness score *C* < 0.5.

### Demographics of patients

More reports were received for females (61.96%, *n* = 5228) than for males (33.05%, *n* = 2789) (Table 4). Sex was not indicated on 421 (4.9%) of the reports. The one-way ANOVA test showed a statistically significant difference in the completeness scores between sexes *F* (2, 8435) = 195.983, *p* ≤ 0.001. The difference between the completeness scores for males (*M* = 0.471, s.e. = 0.004) and females (*M* = 0.465, s.e. = 0.003) had no practical significance *d* ≤ 0.2. Reports that did not indicate the sex of the patient (4.99%, *n* = 421) suffered a 30% vigiGrade penalty and therefore had a large practically significant effect on the completeness score of the report compared with males and females (*d* ≥ 0.8).

**TABLE 4:** Influence of age and sex on completeness of adverse drug reaction reports submitted to South African Health Products Regulatory Authority Pharmacovigilance Unit during the year 2017.

Sex	Number of ADR reports		*M*	s.e.	95% CI	Number of ADR reports per completeness score category							
						< 0.5		0.5 ≥ 0.8		> 0.8		Total	
	*n*	%				*n*	%	*n*	%	*n*	%	*n*	%
Female	5228	61.96	0.465	0.003	0.459 – 0.470	3111	59.5	1528	29.2	589	11.3	5228	100.0
Male	2789	33.05	0.471	0.004	0.463 – 0.479	1662	59.6	763	27.4	364	13.1	2789	100.0
**Age group**													
Adolescent (12 years–17 years)	128	1.52	0.458	0.018	0.422 – 0.493	82	64.1	27	21.1	19	14.8	128	100.0
Adult (18 years–64 years)	4837	57.32	0.511	0.003	0.506 – 0.517	2507	51.8	1616	33.4	714	14.8	4837	100.0
Child (2 years – 11 years)	125	1.48	0.420	0.018	0.384 – 0.455	79	63.2	39	31.2	7	5.6	125	100.0
Elderly (> 65 years)	976	11.57	0.511	0.007	0.498 – 0.524	546	55.9	261	26.7	169	17.3	976	100.0
Infant (28 days – 23 months)	248	2.94	0.386	0.013	0.360 – 0.411	176	71.0	61	24.6	11	4.4	248	100.0
Neonate (0–27 days)	27	0.32	0.363	0.039	0.286 – 0.440	18	66.7	9	33.3	0	0.0	27	100.0

ADR, adverse drug reaction; s.e., standard error of the mean; CI, confidence interval; M, mean.

There was a statistically significant difference in the completeness scores of reports received from patients of different age categories (*F* [6, 8431] = 245.27, *p* ≤ 0.001). A total of 57.32% (*n* = 4837) and 11.57% (*n* = 976) of the reports received were for adult and elderly patients, respectively. The age of the patient was missing on 24.85% (*n* = 2097) of the reports and suffered a 30% vigiGrade penalty. It had a large practically significant effect if the age of the patient was missing (*d* ≥ 0.8). The completeness scores of reports of adult (*M* = 0.511, s.e. = 0.03) and elderly (*M* = 0.511, s.e. = 0.007) patients were higher than for infants (*M* = 0.386, s.e. = 0.013) and neonates (*M* = 0.363, s.e. = 0.039) and the differences in the mean completeness score have a medium to large practical effect (0.5 ≥ *d* ≤ 0.8).

### Seriousness of the reports

The suspected reaction was reported as fatal in 12.47% (*n* = 1052) (Table 5) and serious in 55.87% (*n* = 4714) of the reports. There was a statistically significant difference in the completeness of reports with fatal suspected reactions compared with those with non-fatal suspected reactions (*F* [1, 8436] = 329.429, *p* ≤ 0.001). Where the reactions were fatal (*M* = 0.570, s.e. = 0.003) compared with non-fatal or not indicated (*M* = 0.440, s.e. = 0.007) it had a medium to large practically significant effect (0.5 ≥ *d* ≤ 0.8) on the completeness score of the report. The vigiGrade completeness score of 28.4% (*n* = 299) of the reports where the reactions were fatal was > 0.8 and can be classified as well-documented.

**TABLE 5:** Completeness scores according to seriousness of the case.

Fatal	Number of ADR reports		*M*	s.e.	95% CI	Number of ADR reports per completeness score category							
						< 0.5		0.5 ≥ 0.8		> 0.8		Total	
	*n*	%				*n*	%	*n*	%	*n*	%	*n*	%
No or Not indicated	7386	87.53	0.440	0.003	0.435 – 0.445	4623	62.6	2109	28.6	654	8.9	7386	100.0
Yes	1052	12.47	0.570	0.007	0.557 – 0.583	555	52.8	198	18.8	299	28.4	1052	100.0
**Serious†**													
No	68	0.81	0.393	0.027	0.341 – 0.446	54	79.4	12	17.6	2	2.9	68	100.0
Yes	4714	55.87	0.467	0.003	0.461 – 0.474	2920	61.9	1100	23.3	694	14.7	4714	100.0
**Seriousness† criteria**													
Caused hospitalisation	1410	16.71	0.432	0.006	0.420 – 0.443	927	65.7	362	25.7	121	8.6	1410	100.0
Congenital anomaly	6	0.07	0.348	0.089	0.174 – 0.521	4	66.7	2	33.3	0		6	100.0
Death	1033	12.24	0.572	0.007	0.559 – 0.585	540	52.3	196	19.0	297	28.8	1033	100.0
Disabling	42	0.50	0.491	0.033	0.425 – 0.557	23	54.8	10	23.8	9	21.4	42	100.0
Life threatening	289	3.42	0.474	0.013	0.449 – 0.499	173	59.9	10	23.8	53	18.3	289	100.0
Other	1934	22.92	0.436	0.005	0.427 – 0.446	1253	64.8	467	24.1	214	11.1	1934	100.0

ADR, adverse drug reaction; s.e., standard error of the mean; CI, confidence interval; M, mean.

† , 44.13% (*n* = 3724) of the reports did not indicate seriousness criteria.

From Table 5, completeness of ADR reports submitted to the authority is linked to the seriousness of the event. There was a statistically significant difference in the completeness scores according to the different seriousness criteria (*F* [6, 8431] = 58.526, *p* ≤ 0.001). Reports with suspected reactions that resulted in death (*M* = 0.572, s.e. = 0.007), disability (*M* = 0.491, s.e. = 0.033) or were life threatening (*M* = 0.474, s.e. = 0.013) had a medium to large practically significant effect (0.5 ≥ *d* ≤ 0.8) compared with reports with suspected reactions of congenital anomaly (*M* = 0.348, s.e. = 0.089). Amongst the reports with suspected reactions that caused death, 28.8% (*n* = 297) were well-documented (vigiGrade completeness score > 0.8). None of the congenital anomaly reports met the well-documented criteria.

### Outcome

Table 6 contains the frequencies and completeness of ADR according to different outcomes.

**TABLE 6:** Completeness of reports according to outcomes of the adverse drug reactions.

Outcome	Number of ADR reports		*M*	s.e.	95% CI	Number of ADR reports per completeness score category							
						< 0.5		0.5 ≥ 0.8		> 0.8		Total	
	*n*	%				*n*	%	*n*	%	*n*	%	*n*	%
Died	803	9.52	0.619	0.007	0.606 – 0.632	376	46.8	137	17.1	290	36.1	803	100.0
Not recovered	371	4.40	0.680	0.010	0.661 – 0.699	108	29.1	70	18.9	193	52.0	371	100.0
Recovered	797	9.45	0.661	0.007	0.648 – 0.674	262	32.9	173	21.7	362	45.4	797	100.0
Recovering	201	2.38	0.670	0.013	0.644 – 0.696	64	31.8	45	22.4	92	45.8	201	100.0
Unknown	5343	63.32	0.394	0.003	0.389 – 0.399	3654	68.4	1679	31.4	10	0.2	5343	100.0

ADR, adverse drug reaction; s.e., standard error of the mean; CI, confidence interval; M, mean.

The one-way ANOVA test showed a statistically significant difference in the completeness score for reports with different outcomes *F* (5, 8432) = 624.42, *p* < 0.001. The outcome of the ADR was missing in 10.94% (*n* = 923) of the reports and suffered a 30% vigiGrade penalty. This had a practically significant effect (*d* ≥ 0.8) on the mean completeness score when compared with reports where the outcome of the ADR was specified. On 63.32% (*n* = 5343) of the reports, the outcome was classified as unknown, and there was a large, practically significant difference (*d* ≥ 0.8) between the mean completeness score for reports that were classified as unknown (*M* = 0.394, s.e. = 0.003), compared with all the other outcome categories.

### Anatomical Therapeutic Chemical classes

The reports contained 20 438 different suspected drug-reaction pairs for drugs belonging to different ATC classes (Table 7).

**TABLE 7:** Influence of anatomical therapeutic chemical class of the suspected drug on completeness.

ATC	Number of drug-reaction pairs *N* = 20 438		*M*	s.e.	95% CI	Number of drug-reaction pairs per completeness score category							
						< 0.5		0.5 ≥ 0.8		> 0.8		Total	
	*n*	%				*n*	%	*n*	%	*n*	%	*n*	%
A: Alimentary tract and metabolism	2181	10.67	0.390	0.006	0.378 – 0.403	1662	76.2	372	17.1	147	6.7	2181	100.0
B: Blood and blood-forming organs	1332	6.52	0.592	0.008	0.577 – 0.607	731	54.9	298	22.4	303	22.7	1332	100.0
C: Cardiovascular system	2136	10.54	0.481	0.006	0.469 – 0.493	1342	62.8	593	27.8	201	9.4	2136	100.0
D: Dermatologicals	1053	5.15	0.380	0.014	0.353 – 0.406	741	70.4	234	22.2	78	7.4	1053	100.0
G: Genito urinary system and sex hormones	1467	7.18	0.376	0.010	0.356 – 0.396	121	76.4	277	18.9	69	4.7	1467	100.0
H: Systemic hormonal preparations, excluding sex hormones and insulins	500	2.45	0.392	0.019	0.355 – 0.428	385	77.0	90	18.0	25	5.0	500	100.0
J: Anti-infectives for systemic use	4104	20.08	0.469	0.005	0.460 – 0.479	2391	58.3	1375	33.5	338	8.2	4104	100.0
L: Antineoplastic and immunomodulating agents	3201	15.66	0.470	0.006	0.458 – 0.481	1813	56.6	934	29.2	454	14.2	3201	100.0
M: Musculo-skeletal system	807	3.95	0.421	0.015	0.392 – 0.451	574	71.1	178	22.1	55	6.8	807	100.0
N: Nervous system	1592	7.79	0.396	0.008	0.379 – 0.412	1205	75.7	262	16.5	125	7.9	1592	100.0
P: Antiparasitic products, insecticides and repellents	113	0.55	0.330	0.064	0.204 – 0.457	83	73.9	16	14.2	14	12.4	113	100.0
R: Respiratory system	223	1.09	0.410	0.047	0.319 – 0.502	191	85.7	27	12.1	5	2.2	223	100.0
S: Sensory organs	1302	6.37	0.537	0.028	0.482 – 0.592	849	65.2	369	28.3	84	6.5	1302	100.0
V: Various	427	2.09	0.410	0.028	0.355 – 0.465	330	77.3	70	16.4	27	6.3	427	100.0

ADR, adverse drug reaction; s.e., standard error of the mean; CI, confidence interval; M, mean; ATC, anatomical therapeutic chemical.

There was a statistically significant difference in the completeness score for reports according to the different ATC classes of the suspected drugs *F* (13, 8424) = 48.727, *p* ≤ 0.001. Reports containing suspected drugs that belong to ATC Class B (blood and blood-forming products) had the highest mean completeness score (*M* = 0.592, s.e. = 0.008), and there was a practically significant difference between the completeness score of Class B compared with Classes D, G, H, M, N, P, R and V (*d* ≥ 0.8).

## Discussion

Lack of essential information in ADR reports has been an obstacle in the quality management of signal detection, rendering ADR reports futile.^7,10^ Several tools have been developed and used to measure and enhance the completeness of ADR reports. These include the vigiGrade completeness score, EudraVigilance feedback report, clinical documentation tool, and the quality of ADR reports algorithm.^9,20^ In this study, the authors used the vigiGrade completeness score to assess the completeness of ADR reports received by the SAHPRA during 2017, as it is the tool that was designed to measure the level of clinically relevant information in ICSRs in VigiBase^®^. The main use of vigiGrade is to communicate data quality to member countries of the WHO-PIDM.^21^

Our results indicated that only 11.3% (*n* = 953) of the reports analyzed are considered to be well-documented (*C* > 0.8). This is in line with the findings of Bergvall et al., who used VigiGrade to calculate the completeness score for 3.3 million reports in VigiBase^®^ between 2007 and 2012 and found that only 13% of global reports in VigiBase^®^ were considered well-documented and achieved a completeness score > 0.8.^9^ This indicates that more than half of the reports received did not have the essential and important information required for signal detection. The mean completeness score of the reports analyzed in this study is 0.456 (s.d. = 0.22), which is aligned with the findings made by WHO.^9^ This indicates poor quality of reports, which may be explained by the fact that the study included both serious and non-serious reports. However, there are countries such as Japan where 49.5% of reports had a vigiGrade completeness score above 0.8 and were classified as well-documented reports.

### Completeness of reports for different categories of primary reporters

Physicians reported the most (38.6%), followed by consumers or non-HCPs (28.6%) and other HCPs (25.78%), while pharmacists submitted only 4.3% of reports. Aagaard et al. reported that 75% of reports came from physicians compared with 13% and 11% for other HCPs and consumers, respectively.^22^ Thiessard et al. reported 91% of reports coming from physicians and 5% from pharmacists.^23^ In this study physicians submitted the highest number of reports, but when compared with the other studies, it was not such a convincing majority that may suggest underreporting by South African physicians. Physicians are exposed to serious ADRs in hospital settings and have a clinical background about patients’ medical conditions and therefore they might have higher reporting rates when compared with other reporter types. However, the reporting rates of serious ADRs per primary reporter category were not determined. In this study, the completeness of reports submitted by consumers or non-HCPs did not have a practically significant difference compared with reports submitted by physicians, pharmacists and other HCPs (*p* ≤ 0.001; *d* ≤ 0.2). This indicates that the completeness of reports from consumers is comparable to those submitted by HCPs. Rolfes et al. had previously indicated that HCPs and consumers reported clinical information on a comparable level.^24^ A total of 61% of the reports submitted by physicians had a completeness score < 0.5. Only 9.6% of their reports were considered well-documented, whereas 11.1% of the reports submitted by consumers or non-HCPs are well-documented. However, this finding is of interest as it is expected that physicians should submit reports with more clinical data than consumers because they have access to patients’ complete profiles and medical training.

### Missing information in the case reports

Time-to-onset is considered the most essential information in causality assessment because it indicates the temporal relation between the reaction and the suspect drug. Therefore, its omission in the reports accounts for a 50% vigiGrade penalty. The crucial information to calculate the time-to-onset of the reaction was not provided for 52.8% of the drug-reaction pairs, which means it is impossible to evaluate the causal relationship between the drug and the reaction and possibly detect safety signals from these reports. These findings correspond with a study conducted on the Japanese Adverse Event Report database using VigiGrade^®^ in which time-to-onset could not be calculated in 41.2% cases received from the pharmaceutical companies.^25^ Time-to-onset is determined based on the start date of the drug and the onset date of the reaction. The start date of the suspected drug was missing in 52.6% of cases, while the onset date of the reaction was missing in 5.2% of cases. Lack of awareness, particularly in maintaining ADR quality reporting and complacency by HCPs in accepting standards for ADR reporting, maybe some of the reasons behind 52.8% where time-to-onset cannot be determined.^26^ Other factors that can explain why the onset date of the drug is omitted is that patients cannot remember when they started their treatment, while HCPs may not have access to patient records at the time of reporting. Therefore, it is recommended to use an electronic health records system (EHRS) where these data are captured and accessed when required. The use of EHRS will eliminate recall bias by patients. The use of an EHR system has been studied and confirmed to be an additional source of information in characterising drug safety.^27^ However, in a systematic review by Katatura and Cilliers the authors identified social, technical and environment barriers to the implementation of EHRS in African countries, which included a ‘lack of supporting infrastructure, user training and commitment, political influence or strategy, legislation and regulations and the lack of a framework for implementation and management of EHRs’. South Africa will have to address these barriers to establish a EHRS to track and manage patients in the planned National Health Insurance scheme.^28^ To our knowledge ADR reporting has not been built into any dispensing or practice software used in South Africa.

Information regarding the dose of the medicines was missing in 50.3% of cases, while indication for the suspect drugs was missing in 16% of the drug-reaction pairs. As highlighted by Brajovic et al., knowing the reason for taking the suspect drug is an important factor in determining the significance of the reaction.^27^

Action taken with the suspect drug was missing in 15% of drug-reaction pairs, while 10.9% of reports did not report the outcome of the reaction. Where the outcome was reported, it was classified as unknown on 63.32% of the reports. There was a practically significant difference between mean completeness scores of reports that indicated outcome as unknown or missing compared with reports with other outcome categories (*d* ≥ 0.8). If the outcome is not specified on reports or is reported as unknown, HCPs do not follow up with patients who suffer ADRs. The outcome information is one of the fundamental parameters used in causality assessment.^9^ Information on the outcome of the reaction and the action taken with the suspect drug strengthens the relationship between the suspect drug and the reaction. The lack of these parameters suggests a lack of knowledge by reporters regarding ADR management and the importance of different parameters included in ADR reporting forms. Awareness and skill development programmes on the promotion of quality ADR management and reporting need to be considered to improve the quality of ADR reports. Furthermore, follow-up of patients by HCPs needs to be equally addressed to ensure that complete information is sought from the patients.

This study revealed that 55.9% of the reactions were serious, of which 12.5% were fatal. This corresponds with a Danish study, which indicated that 52% of their reports were serious.^22^ In line with the findings by Durrieu et al., results of this study have indicated an association between the seriousness of the reaction and the completeness of the report.^10^ If the reaction was fatal, life-threatening or resulted in disability, it had a medium to large practically significant effect on the completeness scores of the reports (*p* < 0.001; 0.5 ≥ *d* ≤ 0.8). This indicates that reporters are more concerned about serious reactions, ensuring that reports concerning these reactions are well-documented. Serious cases are often hospitalised, and it is expect that they will have better follow-up and that reporters will have more complete records available while reporting.

A statistically significant difference in completeness score for reports of different ATC classes (*p* < 0.001) was observed. Suspect drugs belonging to ATC class B (blood and blood-forming products) contributed 6.5% of the total number of drugs reported and had the highest mean completeness score of 0.59 compared with the other groups (*p* ≤ 0.001); (*d* ≥ 0.8). Blood and blood-forming agents are used to treat life-threatening medical conditions such as stroke, which may explain why these reports have a higher completeness score than other ATC classes. This may further strengthen the association between the seriousness of the reaction and the completeness of the report.

Age and sex are considered important parameters in causality assessment because they indicate whether the suspect drug was prescribed appropriately or not. We found that the completeness score of reports improved with age, similar to a study conducted on the Saudi Food and Drug Authority PV system.^29^ The completeness score for reports of adults and the elderly patients was higher than those of infants and neonates, and the difference has a medium to large practical significance (*p* < 0.001; 0.5 ≥ *d* ≤ 0.8). This might be because adults and elderly patients can clearly explain their experiences compared with infants and neonates who rely on the caregiver. Furthermore, 52% of the reports were for adults, followed by elderly patients with 11%, while reports for infants and neonates were about 3% and less than 1%, respectively. Increasing co-morbidities and polypharmacy may have contributed to an increased number of reports received for adults and elderly patients.

### Strengths and limitations

This was the first study that has been conducted to evaluate the completeness of South African ADR reports submitted to VigiBase^®^. It is important that HCPs are made aware of the completeness of reports and how it impacts the ability of the SAHPRA to conduct causality assessment and detect signals to ultimately improve patient safety. The results of this study can be utilised to improve training of reporters and technical staff who work in the PV unit and are responsible for data capturing and data verification.

However, the present results are only for reports received in a 1-year study period, and it is not intended to be generalised beyond South Africa. A follow-up study should be conducted to evaluate ADR reporting trends over time. The completeness score of each report is calculated automatically. Therefore, a limitation of this study is that it could not be assessed, which specific information was missing on the reports submitted by different HCPs categories. This study used data downloaded from the VigiBase^®^, and it does not indicate the sender of the report (i.e. MAHs, clinical trials, HCPs or non-HCPs) or the report reporting method (e.g. Med Safety App, e-reporting or paper form). The authors were, therefore, unable to assess the completeness by the sender of the reports. The preferred method to report could also not be determined from the data. A more in-depth analysis could be performed to distinguish the proportion of missing information according to sender and primary reporter and to compare the completeness of ADR reports between MAHs and HCPs.

## Conclusion

This study’s analysis indicates that the completeness of ADR reports submitted to SAHPRA is low and requires immediate intervention to improve the quality of reports. It was found that the completeness of reports submitted by consumers is comparable to those submitted by HCPs, which confirms that consumers have a potential role to play within PV. It is therefore imperative that consumer reporting is encouraged, explored and supported in the country. Multiple measures to improve ADR reporting and quality are recommended, particularly for HCPs. These may include using digital reporting tools or EHRS and increasing awareness for HCPs and the public on ADR reporting and reporting quality. The inclusion of a section in the PIL explaining how and where to submit an ADR is also recommended.
